# Comparing the Effectiveness of Low-Level Laser Therapy and Topical Steroid Therapy Combination Regimen With Routine Topical Steroid Therapy in the Management of Oral Lichen Planus Symptomatic Patients

**DOI:** 10.7759/cureus.44100

**Published:** 2023-08-25

**Authors:** Kalagi G Panchal, Ekta Gupta, Amit Kumar, P V Samir, G S Devika, Vijaya Awasthi, Ramanpal Singh

**Affiliations:** 1 Department of Dentistry, Government Dental College and Hospital, Ahmedabad, IND; 2 Department of Orthodontics and Dentofacial Orthopedics, Siddhpur Dental College and Hospital, Patan, IND; 3 Department of Dentistry, All India Institute of Medical Sciences, Patna, IND; 4 Department of Pedodontics and Preventive Dentistry, Kalinga Institute of Dental Sciences, Kalinga Institute of Industrial Technology (KIIT), Bhubaneswar, IND; 5 Department of Pharmaceutical Analysis, Cherraans College of Pharmacy, Coimbatore, IND; 6 Department of Oral Medicine and Radiology, Gentle Dental Care, Jabalpur, IND; 7 Department of Oral Medicine and Radiology, New Horizon Dental College and Research Institute, Bilaspur, IND

**Keywords:** burning sensation, pain, oral lichen planus, topical steroids, lllt

## Abstract

Background: For symptomatic oral lichen planus (OLP), a wide range of therapeutic approaches have been suggested. To minimize discomfort and symptoms among individuals with symptomatic OLP, extensive therapy is frequently needed. Therefore, finding a new therapeutic approach that may effectively manage OLP's symptoms and signs while having few adverse effects continues to be a difficult task. Recently, low-level laser therapy (LLLT) has become a popular alternative therapy option for OLP with no serious side effects.

Aim:The present research was designed to compare the effectiveness of a combination regimen of LLLT in addition to topical steroids with routine topical steroid therapy separately in order to manage patients with bothersome OLP with an extended period of follow-up.

Materials and methods: In our trial, 60 patients were chosen and given sequential numbers as they signed up to take part. The participants were divided randomly into two categories: category A (LLLT plus topical steroids) and category B (only topical steroids). The data were entered into the aforementioned prepared case template after receiving informed consent. The aforementioned prepared case template included the following criteria for evaluating the result of the treatment: pain, recurrence, burning sensation, clinical remission, and size of the lesion. Applying the visual analog scale (VAS), pain, as well as burning sensations, were assessed in both categories. With the aid of the Electronic Digital Vernier Caliper (Mitutoyo, China), these individuals were assessed for the dimension of the lesion.

Results: The pain score on day 21 of intervention in category A was 2.5, while it was 4.63 in category B. The difference in findings was significant statistically at day 21 (p = 0.0032). The pain score on day 28 of intervention in category A was 1.3, while it was 3.0 in category B. The difference in findings was significant statistically at day 28 (p = 0.003). The pain score was greater in the control category as compared to the intervention category. The burning sensation score on day 21 of intervention in category A was 2.5, while it was 4.5 in category B. The difference in findings was significant statistically (p = 0.0024). The burning sensation score at the follow-up phase on day 45 of intervention in category A was 1.1, while it was 3.4 in category B. The difference in findings was significant statistically (p = 0.002).

Conclusion: Newer therapeutic techniques are becoming accessible to oral specialists for controlling oral mucosal disorders as a result of evolving dental trends. The gold standard for treating people with symptomatic OLP continues to be topical corticosteroids. The therapeutic advantages of topical corticosteroids, however, are considerably outweighed by their complementary effect when paired with newer treatment methods like LLLT.

## Introduction

The cause of the pervasive, mucocutaneous condition referred to as oral lichen planus (OLP) is uncertain. The epidermis and oral mucosa are both affected, and it can happen either simultaneously or separately. The buccal mucosa is the most frequently afflicted oral mucosal region; however, the gingiva, tongue, and labial mucosa can all be influenced [1]. According to reports, the frequency of OLP in the population of adults, in general, is 1.27% worldwide. It affects 1.5% of Indians and is more common in females between the ages of 30 and 60 years [2,3].

A wide range of therapeutic approaches have been suggested for symptomatic OLP, including topical and systemic corticosteroids, surgery, photochemotherapy, azathioprine, antimalarials, topical and systemic retinoids, and topical cyclosporine. To minimize discomfort and symptoms among individuals with symptomatic OLP, extensive therapy is frequently needed. Therefore, finding a new therapy approach that may effectively manage OLP's symptoms and signs while having few adverse effects continues to be a difficult task.

Recently, low-level laser therapy (LLLT) has become a popular alternative therapy option for OLP with no serious side effects [4,5]. The majority of patients have experienced considerable improvement in their clinical symptoms with this painless, harmless therapy method [6]. On the oral mucosa, it has been discovered that LLLT produces biostimulating effects. Additionally, it enhances the healing of tissues by reducing inflammation [7].

In order to manage patients with bothersome OLP with an extended period of follow-up, the present research was designed to compare the effectiveness of a combination regimen (LLLT in addition to topical steroids) with routine topical steroids separately.

## Materials and methods

Study design and sample size

It was randomized, single-blind research. The study included 60 patients, aged between 18 and 60, of either gender (40 females, 20 males). Patients with erosive OLP who had been physically and histopathologically diagnosed were chosen for the research. The ethical clearance was obtained from New Horizon Dental College and Research Institute, Bilaspur, India with IRB number NHDCRI/2023/MDS/OMR/19-ECC.

The sample size was calculated in the following manner: n = (z)2 p (1 - p) / d2, where n = sample size, z = level of confidence according to the standard normal distribution (for a level of confidence of 95%, z = 1.67), p = the estimated proportion of the population that presents the characteristic (when unknown, we use p = 0.3), d = tolerated margin of error (for example, we want to know the real proportion within 5%). Using the above formula, a minimum sample size of 59.4 (equivalent to 60) was calculated.

Inclusion criteria include patients 18 years of age and older who have symptomatic erosive OLP that has been empirically and histopathologically proven and who complete a written authorization and freely agree to participate in the study. Exclusion criteria include patients with numerous OLP lesions, women who are expecting or nursing, patients receiving treatment with steroids for any other purpose as well as those using any medications that may cause a lichenoid reaction, people who have findings of dysplasia in their specimen of biopsy, patients who entered the research within a month after receiving OLP treatment, individuals with any underlying illnesses, and any lesions close to an amalgam restoration or other metallic restoration.

Randomization

In our trial, 60 patients were chosen and given sequential numbers as they signed up to participate. The participants were divided into two categories: intervention (category A) and control (category B), according to the two types of intervention.

Category A: LLLT + Topical Steroid (n = 30)

Category B: Topical steroids (n = 30)

Allocation secrecy and single blinding

The first and ongoing assessments of each participant were completed by the lead investigator. In order to reduce bias, the researcher was unaware of whether the individuals belonged to the experimental category or the control category. Another researcher, who was kept unaware of the individual's symptoms and signs and the information acquired in the case template by the main researcher, executed the treatment measures (LLLT).

Clinical evaluation

The data were entered into the aforementioned prepared case template after receiving informed consent. The aforementioned prepared case template included the following criteria for evaluating the result of the treatment: pain, recurrence, burning sensation, clinical remission, and lesion size. Applying the visual analog scale (VAS), pain, as well as burning sensations, were assessed in both categories. With the aid of the Electronic Digital Vernier Caliper (Mitutoyo, China), these individuals were assessed for the dimension of the lesion corresponding to the region of exposure and were graded using the guidelines suggested by Piboonniyom et al. [8]: 0 means there is no lesion, 1 indicates that there is a lesion and that it is less than 1 cm^2^, 2 indicates the presence of a lesion that ranges in size from 1 to 3 cm^2^, 3 indicates the presence of a lesion that is larger than 3 cm^2^. Lesions were measured based on the area covered by length and breadth in cm^2^, and it was an average of the size bilaterally.

According to the criteria provided by Piboonniyom et al., the full clinical remission of the lesions is characterized as the complete elimination of the condition from the initial site [8]. Patients who completely clinically resolved their lesions within the research's active period were instructed to discontinue treatment, and they had follow-up for an additional two months (at least once every 15 days) to look for any repetition, which was also examined. It was defined as the return of the signs and symptoms at the same site after complete remission; if the recurrence was present, it was recorded in proforma. Recurrence was referred to as the reappearance of symptoms and signs at the same location following complete eradication; if it occurred, it was noted on the case sheet.

Interventions in both categories

**Figure 1:**
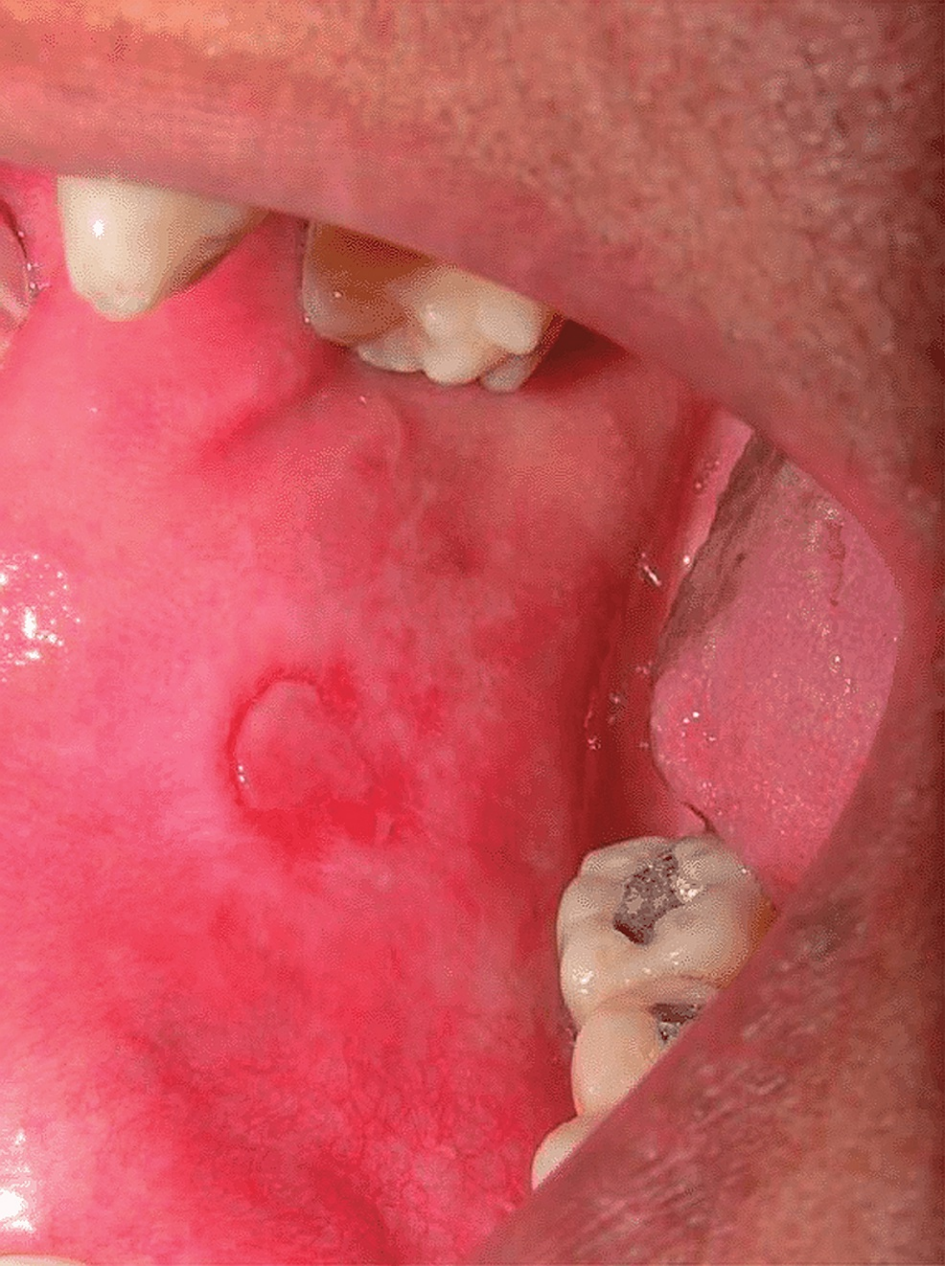
Preoperative lesion

**Figure 2:**
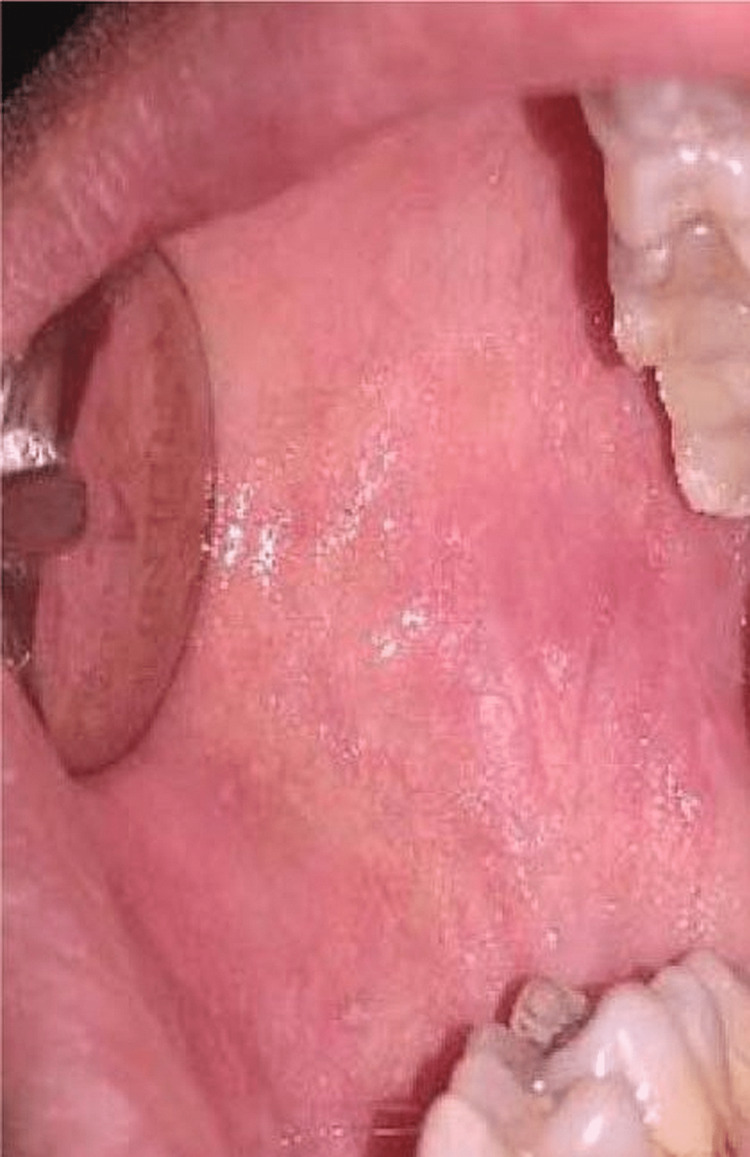
Postoperative lesion resolution after therapy

Both sets of participants (category A and category B) received topical steroid treatment, which included external 0.1% triamcinolone acetonide ointment (application five times daily on the afflicted region for a period of 28 days or until the lesion recovers, whatever is sooner) (Kenacort TM 0.1% Abbott, India). Patients in the experimental category also received LLLT along with the standard therapy. The photon (3W) diode laser used to power the LLLT has the following characteristics: time duration 10 min, power output 0.8-0.9 W, continuous defocused non-contact mode, and wavelength 810 nm, The equipment was utilized as directed by the company that produced it. A 400-m fiberoptic connection and a biostimulation handpiece were used to steer the laser. To evenly spread energy across the lesion's surface, a "spot" method with modest overlapping was employed. The person receiving treatment and all aides (auxiliaries) were instructed to put on special protective spectacles ahead of the treatment with lasers, and all safety laser standards were adhered to. The LLLT was launched with the aforementioned characteristics on day 0. Nine sessions of twice-weekly treatment were required. (Figures 1, 2)

After the person in question was seated comfortably on the couch, the lesion was cleaned of any salivary contaminants by wiping it with sterilized cotton gauze. The area of interest was then carefully covered with a pea-sized gel or 0.1% triamcinolone acetonide oral ointment using a cotton application tip. The gel serves as a surface boundary for the laser's biostimulation. After 28 days, the research's active stage came to a conclusion.

Follow-up phase

On the 45th, 60th, 75th, and 90th days of the follow-up stage after the active period, all the clinical variables were again documented for each patient. In the case of the pro forma, information acquired throughout every active stage and follow-up stage was input. The lesions' photographic documentation was collected at both the baseline and follow-up visits.

Data analysis and statistics

Microsoft Excel 2013 was used to gather, code, and input all the information. The statistical examination was completed using the Statistical Package for Social Software (SPSS) version 22.0 (IBM Corp., Armonk, NY). It was determined that all p values under 0.05 were of statistical significance. Tables were used to convey the findings.

## Results

The pain score at the beginning of the intervention (day 0) in category A was 8.9, while it was 8.8 in category B. The pain score on day 7 of intervention in category A was 6.47, while it was 7.2 in category B. The pain score on day 14 of intervention in category A was 4.47, while it was 5.9 in category B. The difference in pain score was non-significant statistically on days 0, 7, and 14. The pain score on day 21 of intervention in category A was 2.5, while it was 4.63 in category B. The difference in findings was significant statistically at day 21 (p = 0.0032). The pain score on day 28 of intervention in category A was 1.3, while it was 3.0 in category B. The difference in findings was significant statistically at day 28 (p = 0.003). The pain score was greater in the control category as compared to the intervention category C (Table 1).

**Table 1:** Comparison of pain scores between the two categories

Variables	Category A	Category B	t-test	P value
Active phase				
Day 0	8.9	8.8	1.12	0.30
Day 7	6.47	7.2	0.0	0.0
Day 14	4.47	5.9	1.48	0.610
Day 21	2.5	4.63	3.2	0.0032
Day 28	1.3	3.0	2.7	0.003
Passive phase				
Day 45	0.87	3.54	4.61	0.001
Day 60	0.87	3.74	5.57	0.0021
Day 75	1.27	4.0	4.41	0.0046
Day 90	1.47	4.3	4.20	0.0031

During the follow-up phase, the pain score at day 45 of intervention in category A was 0.87, while it was 3.54 in category B. The difference in findings was significant statistically at day 45 (p = 0.001). The pain score at day 60 of intervention in category A was 0.87, while it was 3.74 in category B. The difference in findings was significant statistically at day 45 (p = 0.001). The pain score at day 75 of intervention in category A was 1.27, while it was 4.0 mm in category B. The difference in findings was significant statistically at day 75 (p = 0.001). The pain score at day 90 of intervention in category A was 1.47, while it was 4.3 in category B. The difference in findings was significant statistically at day 75 (p = 0.0031) (Table 1). The reduction in pain score was greater in patients undergoing combination therapy with LLLT and steroids applied topically.

The burning sensation score at the beginning of the intervention (day 0) in category A was 8.9, while it was 8.8 in category B. The burning sensation score on day 7 of intervention in category A was 8.9, while it was 8.8 in category B. The burning sensation score on day 14 of intervention in category A was 5.0, while it was 5.8 in category B. The difference in findings was non-significant statistically on days 0, 7, and 14. The burning sensation score on day 21 of intervention in category A was 2.5, while it was 4.5 in category B. The difference in findings was significant statistically (p = 0.0024). The burning sensation score at the follow-up phase on day 45 of intervention in category A was 1.1, while it was 3.4 in category B. The difference in findings was significant statistically (p = 0.002). The burning sensation score at the follow-up phase on day 60 of intervention in category A was 1.1, while it was 3.8 in category B. The difference in findings was significant statistically (p = 0.004). The burning sensation score at the follow-up phase on day 75 of intervention in category A was 1.1, while it was 4.1 in category B. The difference in findings was significant statistically (p = 0.0004). The burning sensation score at the follow-up phase on day 90 of intervention in category A was 1.3, while it was 4.3 in category B. The difference in findings was significant statistically (p = 0.0003). The reduction in burning sensation score was greater in patients undergoing combination therapy of LLLT and steroids applied topically (Table 2).

**Table 2:** Comparison of the burning sensation scores between the two categories

Variables	Category A	Category B	t-test	P value
Active phase				
Day 0	8.9	8.8	1.11	0.30
Day 7	7.2	7.12	0.1	0.0
Day 14	5.0	5.8	1.38	0.612
Day 21	2.5	4.5	3.2	0.0024
Day 28	1.5	3.0	2.7	0.002
Passive phase				
Day 45	1.1	3.4	3.9	0.002
Day 60	1.1	3.8	3.8	0.004
Day 75	1.1	4.1	4.4	0.0004
Day 90	1.3	4.3	4.2	0.0003

The size of the lesion at the beginning of intervention (day 0) in category A was 1.6 mm, while it was 1.6 mm in category B. The size of the lesion on day 7 of intervention in category A was 1.4 mm, while it was 1.6 mm in category B. The difference in findings was non-significant statistically on days 0 and 7. The size of the lesion on day 14 of intervention in category A was 1.1 mm, while it was 1.6 mm in category B. The difference in findings was significant statistically. (p= 0.024). The size of the lesion on day 21 of intervention in Category A was 0.7 mm, while it was 1.4 mm in category B. The difference in findings was significant statistically. (p= 0.007). The size of the lesion on day 28 of intervention in category A was 0.4 mm, while it was 1 mm in category B. The difference in findings was significant statistically. (p= 0.0084).

During the passive phase, the size of the lesion at day 45 of intervention in category A was 0.4 mm, while it was 1 mm in category B. The difference in findings was significant statistically. (p= 0.0084). The size of the lesion at day 60 of intervention in category A was 0.4 mm, while it was 1 mm in category B. The difference in findings was significant statistically (p= 0.0024). The size of the lesion at day 75 of intervention in category A was 0.3 mm, while it was 1.2 mm in category B. The difference in findings was significant statistically. (p= 0.0008). The size of the lesion at day 90 of intervention in category A was 0.3 mm, while it was 1.4 mm in category B. The difference in findings was significant statistically (p= 0.0006). In this study, the reduction in the size of the lesion was greater in patients undergoing combination therapy of LLLT and steroids applied topically (Table 3).

**Table 3:** Comparison of the lesion's size between the two categories

Variables	Category A	Category B	t-test	P value
Active phase				
Day 0	1.6	1.6	0	0
Day 7	1.4	1.6	1.37	0.50
Day 14	1.1	1.6	2.41	0.024
Day 21	0.7	1.4	2.89	0.007
Day 28	0.4	1	2.83	0.0084
Passive phase				
Day 45	0.4	1	2.83	0.0084
Day 60	0.4	1.1	3.33	0.0024
Day 75	0.3	1.2	3.74	0.0008
Day 90	0.3	1.4	3.87	0.0006

A comprehensive clinical remission was observed in 18 out of 30 patients (about 60%) in the experimental category and in just six out of 30 participants (about 20%) in the control category. So, in the research category, more individuals had lesions in their active stage that completely resolved clinically. There was no further rise in the proportion of patients in either category who had completely resolved their lesions during the whole follow-up stage (Table 4).

**Table 4:** Comparison of the lesions' clinical recovery between the two categories

Variables	Category A	Category B	Chi square test	P value
Active phase				
Day 0	0	0	-	
Day 7	0	0	-	
Day 14	0	0	-	
Day 21	8	2	2.17	0.14
Day 28	18	6	6	0.026
Passive phase				
Day 45	18	6	6	0.026
Day 60	18	4	8	0.009
Day 75	16	2	7.78	0.006
Day 90	16	2	7.78	0.006

There was no recurrence at day 45 in both category A or category B. There was a recurrence in two patients in the control category, while there was no recurrence in the intervention category at day 60. The findings were significant. There was recurrence in four patients in the control category, while there was recurrence in four patients in the intervention category at day 75. There was recurrence in four patients in the control category, while there was recurrence in two patients in the intervention category at day 90. The difference in findings was, however, non-significant statistically at days 60, 75, and 90 of follow-up (Table 5).

**Table 5:** Comparison of the recurrence of the lesion between both the categories

Variables	Category A	Category B	Chi square test	P value
Passive phase				
Day 45	0	0	-	-
Day 60	0	2	0	0
Day 75	4	4	0.39	0.65
Day 90	2	4	0.39	0.65

## Discussion

OLP is a long-lasting inflammatory condition that damages the mucous lining of the mouth and palate [8,9]. It is an autoimmune condition caused by T cells in which cytotoxic CD8+ T cells cause cells of the basal layer of the oral mucosa to undergo apoptosis. In the oral mucosa, lichen planus conditions typically persist longer than they do on the skin. Lichen planus may also return even after entirely disappearing. Lichen planus frequently causes the oral mucosa to become dark brown as it cures [10,11]. Certain drugs, mouth injuries, infections, or allergies brought on by substances like dental material can all cause OLP. One of the factors contributing to the lesion's resurgence or severity is stress. In order to manage patients with bothersome OLP with an extended period of follow-up, the present research was designed to compare the effectiveness of a combination regimen (LLLT in addition to topical steroids) with routine topical steroids separately.

In this study, the reduction in pain score was greater in patients undergoing combination therapy with LLLT and topical steroids. The pain score on day 21 of intervention in category A was 2.5, while it was 4.63 in category B. The difference in findings was significant statistically at day 21 (p = 0.0032). The pain score on day 28 of intervention in category A was 1.3, while it was 3.0 in category B. The difference in findings was significant statistically at day 28 (p = 0.003). The pain score was greater in the control category as compared to the intervention category.

During the follow-up phase, the pain score at day 45 of intervention in category A was 0.87, while it was 3.54 in category B. The difference in findings was significant statistically at day 45 (p = 0.001). The pain score at day 60 of intervention in category A was 0.87, while it was 3.74 in category B. The difference in findings was significant statistically at day 45 (p = 0.001). The pain score at day 75 of intervention in category A was 1.27, while it was 4.0 mm in category B. The difference in findings was significant statistically at day 75 (p = 0.001). The pain score at day 90 of intervention in category A was 1.47, while it was 4.3 in category B. The difference in findings was significant statistically at day 75 (p = 0.0031).

According to Laxmi et al. [9], El Shenawy et al. [10], Rezaei et al. [11], and Suman et al. [12], patients who received LLLT experienced statistically significant improvements in their pain and burning sensation scores as well as a statistically important reduction in the overall dimension of the lesion. OLP, a widespread mucocutaneous disorder, has an unknown etiology. Both the oral mucosa and epidermis can be impacted, and this can happen simultaneously or separately. The buccal mucosa is the area of the oral mucosa that is most usually affected; however, the gingiva, tongue, and labial mucosa can also be affected [13]. According to statistics, 1.27% of adults globally experience OLP on a regular basis. 1.5% of Indians are afflicted, and females between the ages of 30 and 60 years are the most frequently affected [14,15].

The reduction in burning sensation score was greater in patients undergoing combination therapy with LLLT and steroid application tropically. The burning sensation score on day 21 of intervention in category A was 2.5, while it was 4.5 in category B. The difference in findings was significant statistically (p = 0.0024). The burning sensation score at the follow-up phase on day 45 of intervention in category A was 1.1, while it was 3.4 in category B. The difference in findings was significant statistically (p = 0.002).

In this study, the reduction in the size of the lesion was greater in patients undergoing combination therapy with LLLT and steroid application topically. The size of the lesion on day 14 of intervention in category A was 1.1 mm, while it was 1.6 mm in category B. The difference in findings was significant statistically (p= 0.024). The size of the lesion on day 21 of intervention in category A was 0.7 mm, while it was 1.4 mm in category B. The difference in findings was significant statistically (p= 0.007). The size of the lesion on day 28 of intervention in category A was 0.4 mm, while it was 1 mm in category B. The difference in findings was significant statistically (p= 0.0084).

In their research, Dillenburg et al. [15] and Cafaro et al. [16] demonstrated that all lesions in the patients who had LLLT had completely cleared up clinically. More patients experienced full clinical remission of the lesions when LLLT was combined with the surface steroid treatment than when the surface steroid treatment was used alone. There was a recurrence in two patients in the control category, while there was no recurrence in the intervention category at day 60. There was recurrence in four patients in the control category, while there was recurrence in four patients in the intervention category at day 75. There was recurrence in four patients in the control category, while there was recurrence in two patients in the intervention category at day 90. Recurrence was lower with LLLT plus topical steroids. The difference in findings was, however, non-significant statistically at days 60, 75, and 90 of follow-up. Multiple studies by Kollner et al. [17], Passeron et al. [18], Trehan and Taylor [19], and others documented the lesion recurring in a relatively brief period of time.

Extensive therapy is typically required for patients with symptomatic erosive OLP to reduce discomfort and symptoms. Therefore, it remains challenging to identify a novel therapeutic strategy that could manage the symptoms and signs of OLP. Low-level laser treatment (LLLT), a prominent alternative therapy option for OLP without any negative side effects, has recently gained popularity [20-23]. With this painless, safe therapy, the majority of patients have seen a significant improvement in their clinical problems [24,25]. It has been found that LLLT exerts biostimulating effects on the oral mucosa. Additionally, by lowering inflammation, it improves tissue recovery. Despite the apparent absence of severe complications, it remains imperative to acknowledge that any therapeutic intervention, including LLLT, may not be entirely devoid of potential drawbacks. Previous research and clinical experience have highlighted the potential for mild and transient adverse reactions, such as localized discomfort, mild irritation, or temporary sensitivity in some cases. Therefore, while LLLT offers promising therapeutic potential, it is prudent for clinicians and researchers to maintain vigilance and diligently monitor for any unforeseen or subtle adverse effects that could arise from its application, thereby ensuring the holistic assessment of its safety profile and enabling informed clinical decision-making.

The study limitations include additional research that must be conducted using a larger sample size, with post-treatment histopathological analysis. The current study had an extended period of follow-up of up to 45 days. However, considering the chronic nature of OLP, it would be beneficial to conduct studies with longer follow-up periods to assess the long-term efficacy and recurrence rates of the combination therapy. This would provide more comprehensive information on the sustained benefits of LLLT in managing OLP symptoms. Further research is needed to optimize treatment protocols and compare different therapeutic approaches. By addressing these future recommendations, oral specialists and researchers can continue to improve the management of OLP, minimize discomfort for patients, and develop more effective and targeted treatment strategies.

## Conclusions

Newer therapeutic techniques are becoming accessible to oral specialists for controlling oral mucosal disorders as a result of evolving dental trends. The gold standard for treating people with symptomatic OLP continues to be topical corticosteroids. The therapeutic advantages of topical corticosteroids, however, are considerably outweighed by their complementary effect when paired with newer treatment methods like LLLT.

## References

[1] Derikvand N, Ghasemi SS, Moharami M, Shafiei E, Chiniforush N (2017). Management of oral lichen planus by 980 nm diode laser. J Lasers Med Sci.

[2] Carrozzo M, Francia Di Celle P, Gandolfo S (2001). Increased frequency of HLA-DR6 allele in Italian patients with hepatitis C virus-associated oral lichen planus. Br J Dermatol.

[3] Bez C, Hallett R, Carrozzo M (2001). Lack of association between hepatotropic transfusion transmitted virus infection and oral lichen planus in British and Italian populations. Br J Dermatol.

[4] Gangeshetty N, Kumar BP (2015). Oral lichenplanus: etiology, pathogenesis, diagnosis, and management. World J Stomatol.

[5] Chitturi RT, Devy AS, Nirmal RM, Sunil PM (2014). Oral lichen planus: a review of etiopathogenesis, clinical, histological and treatment aspects. JBR J Interdiscip Med Dent Sci.

[6] Sahebjamee M, Arbabi-Kalati F (2005). Management of oral lichen planus. Arch Iran Med.

[7] Mahdavi O, Boostani N, Jajarm H, Falaki F, Tabesh A (2013). Use of low level laser therapy for oral lichen planus: report of two cases. J Dent (Shiraz).

[8] Piboonniyom SO, Treister N, Pitiphat W, Woo SB (2005). Scoring system for monitoring oral lichenoid lesions: a preliminary study. Oral Surg Oral Med Oral Pathol Oral Radiol Endod.

[9] Laxmi GD, Khan M, Johar K, Vijaylakshmi KR, Nikita G (2015). A comparative study on use of diode laser and topical triamcinolone acetonide 0.1% in the management of oral lichen planus. Int J Dent Res.

[10] El Shenawy HM, Eldin AM (2015). A comparative evaluation of low-level laser and topical steroid therapies for the treatment of erosive-atrophic lichen planus. Open Access Maced J Med Sci.

[11] Dalirsani Z, Seyyedi SA (2021). Treatment of Plaque-Like Oral Lichen Planus with CO2 Laser. Indian J Dermatol.

[12] Suman V, Peta N, Bokkasam V, Babu DS, YV. S, Puchalapalli Y (2019). Management of pain in erosive lichenplanus using low-level lasertherapy (LLLT): a report of two cases. Int J Contemp Med Surg Rad.

[13] Ferrara N, Gerber HP, LeCouter J (2003). The biology of VEGF and its receptors. Nat Med.

[14] Thomas M, Augustin HG (2009). The role of the angiopoietins in vascular morphogenesis. Angiogenesis.

[15] Dillenburg CS, Martins MA, Munerato MC (2014). Efficacy of laser phototherapy in comparison to topical clobetasol for the treatment of oral lichen planus: a randomized controlled trial. J Biomed Opt.

[16] Cafaro A, Arduino PG, Massolini G, Romagnoli E, Broccoletti R (2014). Clinical evaluation of the efficiency of low-level laser therapy for oral lichen planus: a prospective case series. Lasers Med Sci.

[17] Köllner K, Wimmershoff M, Landthaler M, Hohenleutner U (2003). Treatment of oral lichen planus with the 308-nm UVB excimer laser--early preliminary results in eight patients. Lasers Surg Med.

[18] Passeron T, Zakaria W, Ostovari N, Mantoux F, Lacour JP, Ortonne JP (2004). Treatment of erosive oral lichen planus by the 308 nm excimer laser. Lasers Surg Med.

[19] Trehan M, Taylor CR (2004). Low-dose excimer 308-nm laser for the treatment of oral lichen planus. Arch Dermatol.

[20] Akram Z, Javed F, Hosein M, Al-Qahtani MA, Alshehri F, Alzahrani AI, Vohra F (2018). Photodynamic therapy in the treatment of symptomatic oral lichen planus: a systematic review. Photodermatol Photoimmunol Photomed.

[21] He Y, Deng J, Zhao Y, Tao H, Dan H, Xu H, Chen Q (2020). Efficacy evaluation of photodynamic therapy for oral lichen planus: a systematic review and meta-analysis. BMC Oral Health.

[22] Rad M, Hashemipoor MA, Mojtahedi A, Zarei MR, Chamani G, Kakoei S, Izadi N (2009). Correlation between clinical and histopathologic diagnoses of oral lichen planus based on modified WHO diagnostic criteria. Oral Surg Oral Med Oral Pathol Oral Radiol Endod.

[23] Thongprasom K, Luengvisut P, Wongwatanakij A, Boonjatturus C (2003). Clinical evaluation in treatment of oral lichen planus with topical fluocinolone acetonide: a 2-year follow-up. J Oral Pathol Med.

[24] Pfütze M, Niedermeier A, Hertl M, Eming R (2007). Introducing a novel autoimmune bullous skin disorder intensity score (ABSIS) in pemphigus. Eur J Dermatol.

[25] Slade GD (1997). Derivation and validation of a short-form oral health impact profile. Community Dent Oral Epidemiol.

